# The Role of Speckle Strain Echocardiography in the Diagnosis of Early Subclinical Cardiac Injury in Cancer Patients—Is There More Than Just Left Ventricle Global Longitudinal Strain?

**DOI:** 10.3390/jcm10010154

**Published:** 2021-01-05

**Authors:** Michal Laufer-Perl, Dan Gilon, Livia Kapusta, Zaza Iakobishvili

**Affiliations:** 1 Department of Cardiology, Tel-Aviv Sourasky Medical Center, Affiliated to the Sackler School of Medicine, Tel Aviv University, Tel Aviv 6423906, Israel; michalpela@gmail.com; 2 Heart Institute, Faculty of Medicine, Hadassah-Hebrew University Medical Center, Hebrew University, Jerusalem 90000, Israel; dangi@ekmd.huji.ac.il; 3 Shamir Medical Center (Assaf Harofeh), Department of Cardiology, Rishon Lezion 7528809, Israel; 4 Pediatric Cardiology Unit, Dana-Dwek Hospital, Tel-Aviv Sourasky Medical Center, Affiliated to the Sackler School of Medicine, Tel Aviv University, Tel Aviv 6423906, Israel; liviak@tlmvc.gov.il; 5 Department of Pediatrics Cardiology, Amalia Children’s Hospital, Radboud University Medical Centre, 6500 HB Nijmegen, The Netherlands; 6 Department of Community Cardiology, Tel Aviv Jaffa District, Clalit Health Fund, and Faculty of Health Sciences, Ben-Gurion University of the Negev, Beer-Sheva 8499000, Israel

**Keywords:** cardiotoxicity, cardio-oncology, speckle tracking, global longitudinal strain, left atrial strain, right ventricle strain, diastolic strain

## Abstract

With the improvement in survival rate, cardiotoxicity has emerged as a significant adverse effect of cancer therapy. Early diagnosis of subclinical cardiac injury may allow the initiation of cardioprotective therapy and preventing the interruption of optimal cancer therapy and the development of irreversible cardiac dysfunction. In this article, we review the role of two-dimensional speckle tracking echocardiography (2D-STE), beyond the common left ventricle global longitudinal strain in the diagnosis of early subclinical cardiac injury in patients treated with cancer therapies.

## 1. Introduction

Over the last several decades, the advance in cancer therapies has led to a significant increase in survival rate, with over 16 million cancer survivors in the United States to date [1]. The improvement in survival has exposed the scope of long-term side effects, with cardiovascular disease (CVD) being a leading cause of morbidity and mortality [2]. Although anthracyclines (ANT) are still considered as the most familiar cardiotoxic drugs [3], monoclonal antibodies against human epidermal growth factor receptor 2 (HER2), vascular endothelial growth factor inhibitors (VEGFi), tyrosine kinase inhibitors (TKI), proteasome inhibitors (PI), immune checkpoint inhibitors (ICIs) and mediastinal radiation have been all recognized to have cardiotoxic effect [4,5]. The most common definition of cardiotoxicity is cancer therapeutics-related cardiac dysfunction (CTRCD) which is based on left ventricular Ejection Fraction (LVEF) evaluation [3,6]. Cardiotoxicity it is not limited solely to left ventricle (LV) dysfunction, but can be expressed as acute coronary syndrome, arrhythmias, valvular disease, pericardial disease, systemic and pulmonary hypertension [5,7].

Numerous studies have shown that detection of early cardiac injury is crucial and may lead to full or partial recovery of the LV function with early intervention, either by adjusting the cancer therapy or by initiating cardioprotective treatment [3,8,9]. Currently, two-dimensional (2D) echocardiography is the method of choice for the evaluation of cardiotoxicity during cancer therapy, using mainly LVEF assessment [6]. LVEF has a number of limitations, including inter- and intra-observer variability, load-dependency and changes evident only after significant myocardial damage occurs. Therefore, there is a need for a more sensitive tool for the detection of small subclinical changes in cardiac function [6,10]. Both three-dimensional (3D) echocardiography and cardiac magnetic resonance (CMR) have improved inter- and intra-observer variability, with higher sensitivity and lower false-negative rate and are therefore more reliable and accurate tools in the assessment of LVEF and cardiac function in cancer patients [11,12]. Despite their advantages both exams are not routinely used for the assessment of patients treated with cancer therapies due to the lack of accessibility, trained staff and relatively high cost.

In recent years, there is a rising use of 2D speckle tracking echocardiography (2D-STE), for the measurement of myocardial deformation as a marker of contractility and elasticity [6]. The use of 2D-STE is feasible, partially by using automated image analysis. However, since 2D-STE strongly depends on image quality, visual check should be performed constantly. Furthermore, 2D-STE is considered angle-independent, comparing to LVEF and in contrast to tissue Doppler imaging [13] and has the ability to differentiate active versus passive movement [14]. Two-dimensional-STE has a number of limitations including load dependency as it is decreases in the presence of decreased preload, increased afterload and increased heart rate [13]. Importantly, cut-off values for myocardial dysfunction are vendor-specific [15]. It is strongly recommended to use the same ultrasound machine and the same 2D speckle-tracking software for longitudinal analysis evaluation and follow up [16]. Recently, vendor-independent software packages (TOMTEC Imaging Systems GMBH, Germany) showed improved correlation between the measured values using different vendors. Currently, global longitudinal strain (GLS) is considered to be a sensitive marker of cardiac injury in cancer patients [6,12,17], yet other 2D-STE utilities should be explored.

In this paper we focus on present and future role of 2D-STE, beyond the commonly used LV GLS, for the early diagnosis of subclinical cardiac injury in patients treated with cancer therapies.

## 2. LV Global Longitudinal Strain

**Figure 1:**
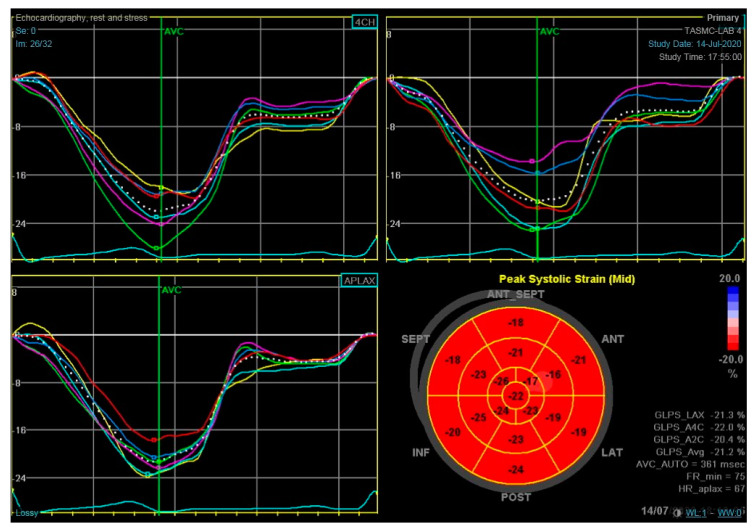
Left ventricle global longitudinal strain measurement GLS was assessed in three apical views, 4 chamber, 2 chamber and 3 chamber views, with 6 segments measured per each view (presented in the different colors), with a total of 18 segments per each exam.

Longitudinal strain is derived from apical images reflecting the changes in the distance between two segments of the heart relative to their baseline distance apart [18] (Figure 1). LV GLS was reported to be a marker of subclinical LV dysfunction with high correlation to CMR measurements [19] and stronger association with prognosis, compared to LVEF, in non-cancer population [14,20]. Other observational studies showed the potential use of LV GLS reduction as a predictor for LVEF reduction in cancer patients [21,22] and in the prevention of the cessation of cancer therapies [23]. The benefit of LV GLS reduction as an early marker for cardiac injury was seen both in patients treated with ANT [24,25], as well as in other cancer populations [17,26], including patients diagnosed with ICIs-related myocarditis [27]. Recently, Pourier et al. [28] showed that strain values decreased during ANT treatment and an ongoing reduction were seen at the latest follow-up (>5 years after therapy with ANT) with preserved cardiac function. Accordingly, it is considered as the optimal parameter for the early detection of subclinical LV dysfunction by the Society of Echocardiography and the European Association of Cardiovascular Imaging [6].

Overall, the accepted normal cutoff value for LV GLS is ≤−18% [12]. A study by Negishi et al. [29] showed that the stronger predictor of CTRCD development was a LV GLS relative reduction of 11% and above during cancer therapy. Whereas relative reduction below 8% is considered not meaningful, a relative reduction above 15% is clinically significant. The latter is supported by the recent position statements [12]. While abnormal baseline or relative changes in LV GLS has been well studied and described as predictors for LV dysfunction and worse prognosis [12], there is little evidence to support initiation of cardioprotective therapy guided by GLS changes [3,30]. The recently published multicenter randomized SUCCOUR trial was designed to compare whether LV GLS versus LVEF-guided approach for cardioprotective therapy initiation will prevent LVEF reduction and CTRCD development [31]. Overall, 307 patients exposed to ANT therapy and had additional heart failure risk, were included. Significant LV GLS relative reduction was determined as ≥12% for the initiation of cardioprotective treatment. At a 1-year follow-up, while no significant change in LVEF was observed between the two arms, fewer patients were diagnosed with CTRCD in the LV GLS-guided group and LVEF was higher. This important trial support for the first time the use of LV GLS for the initiation of cardioprotective therapy and the need for more randomized trials [31].

## 3. LV Circumferential and Radial Strain

Both LV global circumferential strain (LV GCS) (−20.9% to −27.8%, mean −23.3%) and LV radial strain (LV GRS) (35.1% to 59.0%, mean 47.3%) have wide range of normal values [32]. The lower reproducibility of these measurements, mainly LV GRS, makes the identification of changes during or post cancer therapy challenging. Emerging data have shown that LV GCS is decreased during ANT therapy [33] and can predict cardiotoxicity development [34]. In cancer survivors, LV GCS and LV GRS were consistently abnormal, in the presence of normal LVEF. Although a reduction in peak systolic LV GRS of 6% to 17% [34] may indicate early myocardial changes, these changes have been less consistent with the development of LV dysfunction or heart failure comparing to LV GLS [14,34]. A systemic review evaluating 1504 patients during or after cancer chemotherapy concluded that the clinical value of LV GCS and LV GRS in predicting cardiotoxicity is limited and that LV GLS appears to be the preferred measurement [20].

## 4. Multi-Layers Strain

Commonly, the myocardial wall is subdivided into an epicardial, mid-myocardial, and endocardial layers. However, the clinical value of layer-specific assessment is questionable, mainly due to low visibility of the wall for tracking [13]. Although contractile force is greater in the endocardial layer than in the epicardial layer [35], it is also more susceptible to injury due to its higher energy requirements. Previous studies have demonstrated that layer specific strain is more accurate for discriminating the various intensities of transmural scarring compared to CMR [36]. Layer-specific strain analysis provides additional information on disease progression compared to LV GLS alone in cases of heart failure, hypertensive cardiomyopathy, aortic stenosis, and ischemia [35,37]. In a recent study by Chang et al. [38] LV endocardial strain was significantly reduced after three cycles of ANT therapy compared to epicardial strain, despite no significant changes in routine LV systolic and diastolic parameters. The decline in endocardial values prior to LVEF changes suggests that myocardial injury begins from the endocardial layer. However, according to the 2014 European Association of Cardiovascular Imaging (EACVI) expert consensus statement reduction was seen in all three strain layers of the myocardium with no specific preference [6,24]. To date the value of layer-specific strain in cancer patients is considered limited and contradicting [20] and therefore there is no recommendation for its use for surveillance.

## 5. Diastolic Strain

**Figure 2:**
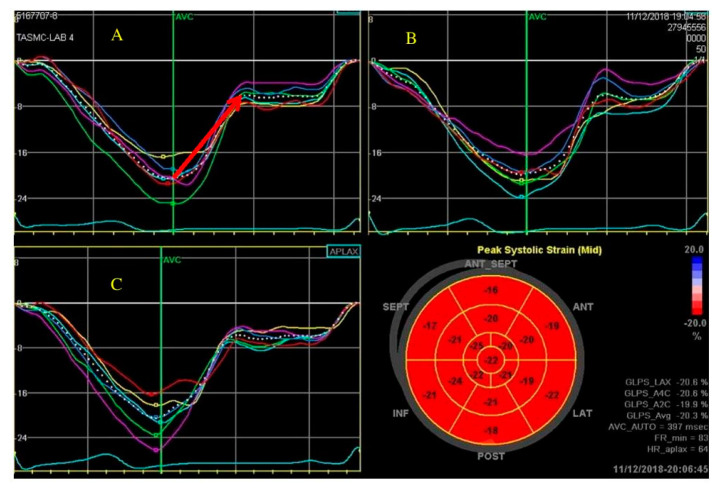
Diastolic strain time (Dst) measurement. Dst was evaluated by measuring manually the time of lengthening (ms) of the myocardium during diastole, from the point of aortic valve closure (AVC) throughout the isovolumic relaxation time and early diastolic, until the plateau of the curve. Dst was assessed in three apical views, 4 chamber (**A**), 2 chamber (**B**) and 3 chamber (**C**) views, with 6 segments measured per each view (presented in the different colors), with a total of 18 segments per each exam. The red arrow shows how the measurement was performed manually for the average of the 6 segments in the 4-chamber view.

Diastolic dysfunction is considered common among cancer patients; yet no clear evidence has shown that it can predict future LV systolic dysfunction [6]. Diastolic strain has the advantages of being less angle dependent and evaluating directly the relaxation of the ventricles rather than indirect measurements of filling pressures. Using 2D-STE for the evaluation of diastolic function, specifically global diastolic strain rate, has been shown to have prognostic value regarding cardiovascular morbidity and mortality in the general population [39,40]. However, information is lacking in cancer patients [41]. A study by Stoodley et al. showed changes in diastolic strain rate among breast cancer patients treated with chemotherapy with association for LV dysfunction development [41]. Recently, Hochstadt et al. evaluated the role of diastolic strain time and slope, measuring manually the lengthening of the myocardium during diastole (Figure 2). Among cancer patients, diastolic strain time and slope showed high correlation to the routine diastolic echocardiography parameters. Furthermore, change in Diastolic strain time and slope was associated with systolic dysfunction development [42,43]. Due to limited data and small trials, diastolic strain is not currently performed as part of the routine evaluation of 2D-STE in cancer population.

## 6. Left Atrial Strain

**Figure 3:**
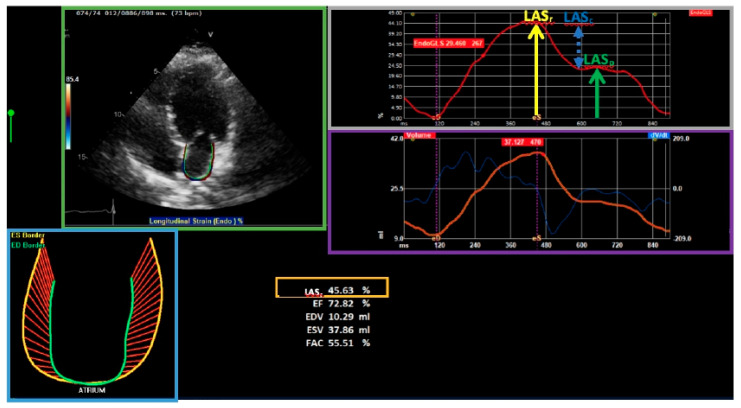
Left atrial strain measurement. Region of interest of the left atrial endocardial border, from the mitral annulus level to the pulmonary veins and/or LA appendage orifice, up to the opposite mitral annulus side were assessed. LASr = left atrial strain during reservoir phase (yellow arrow); LASc = left atrial strain during conduit phase (blue dot arrow); LASp = left atrial strain during atrial contraction phase (green arrow).

The left atrium (LA) plays an important role in the regulation of filling pressure and LV diastolic function. The LA functions as a reservoir to blood returning from the pulmonary veins, as a conduit to blood flowing during early diastole to the LV and as a pump contracting in late diastole (Figure 3). Left atrial strain (LAS) provide a more accurate evaluation of the diastolic function, compared to the routine echocardiographic parameters [44], as determined by invasive measurement (wedge pressure) [45]. LAS evaluation is considered more feasible as it can be calculated from a single apical four-chamber view and is independent to mitral valve pathology. Decreased LAS, with LAS reservoir (LASr) specifically, was found useful in predicting cardiovascular outcomes in healthy and heart failure populations [46,47]. LASr value below 35% is considered as diastolic dysfunction [48]. A recent study conducted on pediatric patients after ANT therapy found a significant decrease in LAS [49]. In contrast, Santoro et al. did not find significant changes in LAS among adult patients during ANT exposure [50]. Furthermore, to date there is no data regarding the predictive value of relative change in LAS on cardiac outcomes.

## 7. Right Ventricle Strain

**Figure 4:**
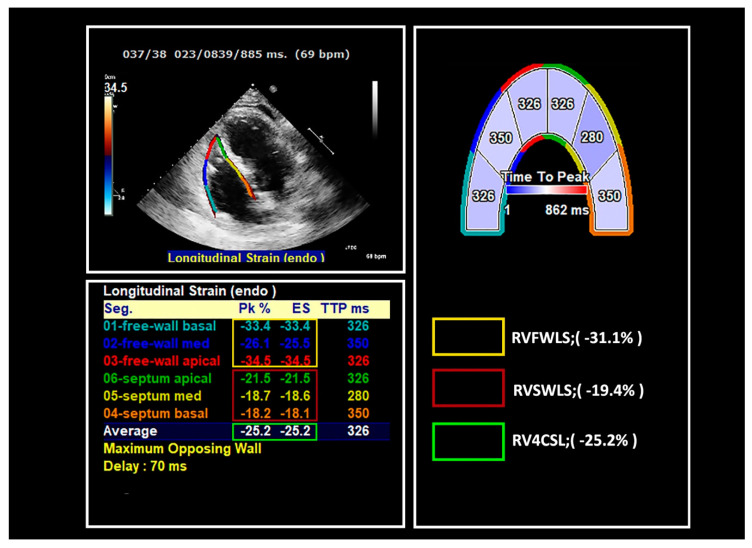
Right ventricle strain measurement. Region of interest of the right ventricle (RV) was defined by the endocardial border at the lateral tricuspid annulus level to the medial tricuspid annulus level. Then, the software automatically divided the apical right chamber views in 6 segments (three separate basal, mid and septal RV free wall strain traces, and three separate basal, mid and septal RV septal wall strain traces) and mathematically calculated RV global 4-chamber contour (RV4CSL) by averaging values of equal segment strain. Peak strain was defined as the peak value during entire heart cycle that may coincide with end-systolic peak or may appear after aortic valve closure. End systole (ES) was defined by the tricuspid valve opening time or by nadir of volume curve as surrogate. Segmental longitudinal strain curves. Note, free-wall strain segments are usually higher than septal strain segments.

There is a rising interest in the use of RV global longitudinal strain (RV GLS) in different populations, with an observation of reduced RV GLS in both preserved and reduced heart failure patients [51] (Figure 4). Data on RV dysfunction in cancer patients remain limited and currently the prognostic value of RV dysfunction is unknown [6]. This may be the result of the inaccurate methods of RV evaluation, including fractional area changes and tricuspid annular plane systolic excursion (TAPSE). A number of studies showed that RV GLS and RV free wall longitudinal strain (RV FWLS) decrease during cancer treatment [52,53]. RV GLS impairment seems to develop almost simultaneously with LV GLS impairment in breast cancer patients. However, the long-term prognostic implications of RV deformation mechanics are unknown. In breast cancer patients receiving ANT, the decline of RV FWLS correlated significantly with the development of dyspnea, independently of LV systolic and diastolic function [54]. Furthermore, in patients with stage III non-small-cell lung cancer under concurrent chemotherapy and radiotherapy, the baseline and change of RV FWLS have been proven to be an independent predictor of all-cause mortality, with worse prognosis in patients presenting with a RV FWLS ≥ 10.1% decrease [55].

The normal cut-off value of RV GLS is considered—18.2% and above [51]. The proposed cutoff value of relative percent decrease RV GLS to identify cardiotoxicity is considered 14.8% and above, similar to that of LV GLS [3].

Clearly, the role of RV is emerging as a marker of cardiac injury in cancer patients and may be at least as frequent as LV injury. However, its prognostic value remains to be proven by large prospective studies.

## 8. Right Atrial Strain

Wright et al. [56] evaluated the correlation between right atrial strain (RAS) measurement and invasive measurement of right atrial pressure (RAP), showing an association with right atrial (RA) size, right ventricle (RV) systolic function and inferior vena cava (IVC) size. Among HF patients, RA conduit and reservoir function are independent predictors of mortality [57]. To the best of our knowledge there is no data regarding the role of RAS as predictor for early acute cardiotoxicity development in cancer patients.

## 9. Conclusions

CV imaging prior to the beginning of cancer therapy provides estimation of preexisting cardiac damage and establishes baseline information for future changes. Currently there are recommendation for baseline and serial LVEF follow-up only among patients treated with ANT and trastuzumab (a type of HER2 targeted therapy) [58], however, these recommendations are not consistently applied. Recently a position paper by the Heart Failure Association (HFA), EACVI and European Society of Cardiology (ESC) recommended a more frequent and personalized echocardiography surveillance according to the specific cancer therapy [12]. However, LVEF screening remains sub-optimal and decision concerning the interruption of potentially lifesaving therapy should not rely solely on LVEF changes.

2D-STE is a reliable and sensitive marker of early cardiac injury with a high availability, low cost, no radiation and no nephrotoxic effect compared to other cardiac imaging modalities. It is recommended to use the same vendor’s machine and software version during serial follow-up examinations [6]. Early reduction in myocardial deformation appears to forecast the development of subsequent cardiotoxicity, with 2D-STE and especially LV GLS being the most consistent parameter [20], even before LV function deteriorates.

While the high value of LV GLS is well recognized, 2D-STE has wide and less familiar modalities, specifically diastolic strain, LAS and RV GLS that are showing a promising role in the diagnosis of early sub-clinical cardiac injury in patients treated with cancer therapies. Future large randomized studies are needed in order to evaluate the role of all 2D-STE modalities in cancer patients, both as a routine surveillance as well as a dictating tool for the beginning of cardioprotective therapy.
